# Effect of ZnSO_4_, MnSO_4_ and FeSO_4_ on the Partial Hydrogenation of Benzene over Nano Ru-Based Catalysts

**DOI:** 10.3390/ijms22147756

**Published:** 2021-07-20

**Authors:** Haijie Sun, Yiru Fan, Xiangrong Sun, Zhihao Chen, Huiji Li, Zhikun Peng, Zhongyi Liu

**Affiliations:** 1 School of Chemistry and Chemical Engineering, Zhengzhou Normal University, Zhengzhou 450044, China; sunhaijie406@zznu.edu.cn (H.S.); FyR158905@sohu.com (Y.F.); sun1398041920@sohu.com (X.S.); huijili@zznu.edu.cn (H.L.); 2 Zhengzhou Tobacco Research Institute of CNTC, Zhengzhou 450001, China; 3 College of chemistry and molecular engineering, Zhengzhou University, Zhengzhou 450001, China; liuzhongyi406@sohu.com

**Keywords:** benzene, partial hydrogenation, cyclohexene, reaction modifier, ZnSO_4_, MnSO_4_, FeSO_4_

## Abstract

Nano Ru-based catalysts, including monometallic Ru and Ru-Zn nanoparticles, were synthesized via a precipitation method. The prepared catalysts were evaluated on partial hydrogenation of benzene towards cyclohexene generation, during which the effect of reaction modifiers, i.e., ZnSO_4_, MnSO_4_, and FeSO_4_, was investigated. The fresh and the spent catalysts were thoroughly characterized by XRD, TEM, SEM, XPS, XRF, and DFT studies. It was found that Zn^2+^ or Fe^2+^ could be adsorbed on the surface of a monometallic Ru catalyst, where a stabilized complex could be formed between the cations and the cyclohexene. This led to an enhancement of catalytic selectivity towards cyclohexene. Furthermore, electron transfer was observed from Zn^2+^ or Fe^2+^ to Ru, hindering the catalytic activity towards benzene hydrogenation. In comparison, very few Mn^2+^ cations were adsorbed on the Ru surface, for which no cyclohexene could be detected. On the other hand, for Ru-Zn catalyst, Zn existed as rodlike ZnO. The added ZnSO4 and FeSO_4_ could react with ZnO to generate (Zn(OH)_2_)_5_(ZnSO_4_)(H_2_O) and basic Fe sulfate, respectively. This further benefited the adsorption of Zn^2+^ or Fe^2+^, leading to the decrease of catalytic activity towards benzene conversion and the increase of selectivity towards cyclohexene synthesis. When 0.57 mol·L^−1^ of ZnSO_4_ was applied, the highest cyclohexene yield of 62.6% was achieved. When MnSO_4_ was used as a reaction modifier, H_2_SO_4_ could be generated in the slurry via its hydrolysis, which reacted with ZnO to form ZnSO_4_. The selectivity towards cyclohexene formation was then improved by the adsorbed Zn^2+^.

## 1. Introduction

**Scheme 1:**
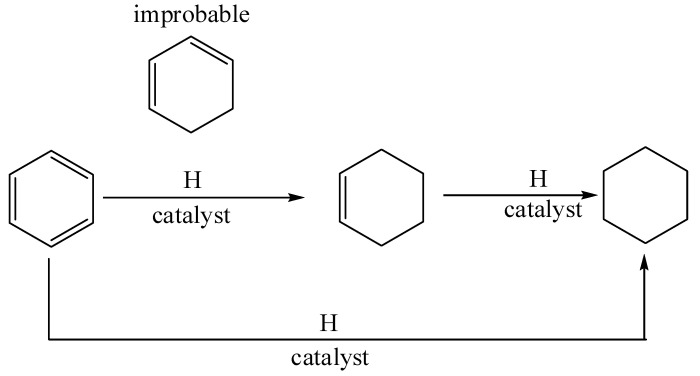
Reaction mechanism for hydrogenation of benzene.

Cyclohexene, a compound with an unstable double bond, can be utilized to synthesize adipic acid, cyclohexanol, and cyclohexanone [1], which are important monomers for the production of nylon 6 and nylon 66. Dehydration of cyclohexanol, dehydrochlorination of halogenated cyclohexane, and Birch reduction methods were traditionally applied for the production of cyclohexene. However, high cost of crude material, side products, and environmental issues are generally addressed, limiting the industrialization of cyclohexene production [2]. In comparison, because of safety, energy conservation, environmental friendliness, and high carbon atom economy, great attention is drawn to the production of cyclohexene via partial hydrogenation of benzene, of which the reaction mechanism is given in Scheme 1. As can been seen in Scheme 1, cyclohexane formation could be done either by direct hydrogenation of benzene or via cyclohexene as an intermediate. It was also reported by Rochin et al. [3] that 1,3-cyclohexadiene could not be detected since diene is extremely unstable due to the further hydrogenation. On the other hand, unfortunately, it is thermodynamically difficult to obtain cyclohexene, since the standard free energy change for cyclohexane formation from benzene hydrogenation is −98 kJ mol^−1^, which is much lower than that for selective hydrogenation to cyclohexene, i.e., −23 kJ mol^−1^ [4]. Therefore, it is of great necessity to develop a suitable catalytic system with high selectivity towards cyclohexene generation.

A reaction modifier is one of the most effective approaches to improve the selectivity towards cyclohexene [5,6,7] by adding one or several compounds into the reaction slurry to modify the surface of catalysts. In general, cyclohexene can hardly be obtained without applying any reaction modifiers over Ru catalysts [8]. ZnSO_4_, FeSO_4_, CdSO_4_, CoSO_4_, NiSO_4_, CuSO_4_, and MgSO_4_ were reported as reaction modifiers for partial hydrogenation of benzene towards cyclohexene formation over Ru-based catalysts, among which ZnSO_4_ and FeSO_4_ were considered the most effective ones. Notably, it is still controversial in regard to the mechanism for the effect of the reaction modifier. For instance, on one hand, Struijk et al. proposed that the more difficult it is to reduce the cations, the higher are selectivity and yield towards cyclohexene generation [9]. Furthermore, Costa et al. proposed that the oxidation of ZnNb_2_O_6_ contributed to catalytic selectivity over Ru catalysts [10], while Spod et al. stated Zn(OH)_3_^−^ played a significant role in improving the selectivity over Ru catalysts [11]. On the other hand, it was reported by Wang et al. that the binding energy of Zn2p_3/2_ over Ru-Zn/m-ZrO_2_ is close to that observed for metallic Zn, declaring that Zn^2+^ was reduced into metallic Zn by the spilled H on the Ru surface [12]. Interestingly, both metallic Zn and Zn^2+^ were observed over Ru-Zn/HAP by Zhang et al. [13]. In general, the effect of Zn can be addressed as follows: (1) the bonding status of hydrogen on the catalyst surface was modified by Zn [14]. The total amount of bonded hydrogen was declined, while the amount of weak bonded hydrogen was enhanced, leading to lower activity towards benzene conversion and higher selectivity towards cyclohexene, respectively; (2) Zn could cover part of the active sites, which is favorable for complete hydrogenation of benzene towards cyclohexane generation [12]; (3) some electrons could be transferred from Zn to Ru, and Ru^δ^^−^ might benefit the formation of cyclohexene [12,13]; (4) the adsorption of cyclohexene on the Ru surface could be retarded by the presence of Zn, hindering the further hydrogenation of cyclohexene to cyclohexane [15]. Generally, these controversies are mainly attributed to the development of technologies for the catalyst characterization. Therefore, for the better design of the Ru-based catalytic system on partial hydrogenation of benzene towards cyclohexene production, it is of great significance to reveal the state of Zn and the effect of reaction additives.

Based on the aforementioned situation, ZnSO_4_, FeSO_4_, and MnSO_4_ were applied for the partial hydrogenation of benzene towards cyclohexene formation over monometallic Ru catalyst and Ru-Zn catalyst. The mechanism in which the reaction modifier affects the catalytic activity of nano Ru-based catalysts was thoroughly investigated by XRD, XRF, TEM, and XPS.

## 2. Results and Discussions

### 2.1. Ru Catalyst

**Figure 1:**
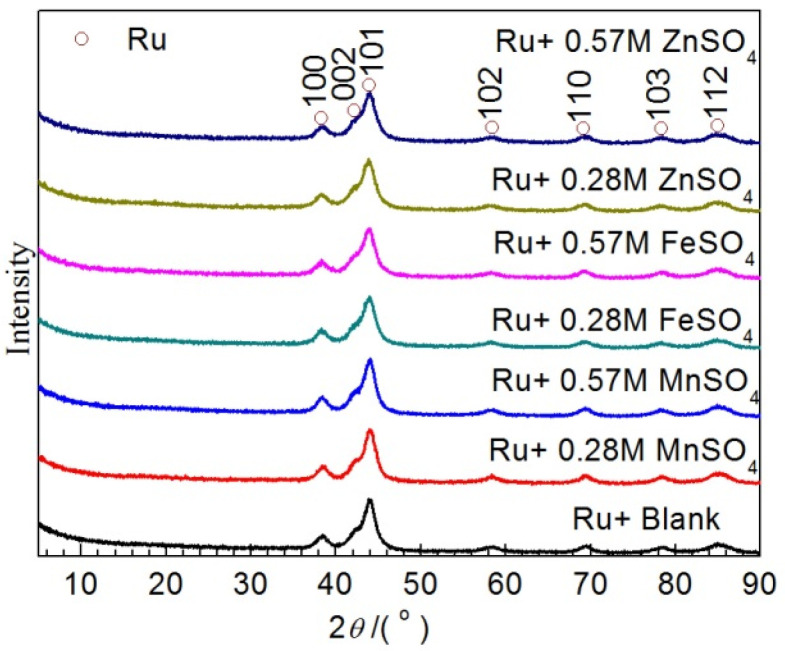
XRD patterns of the monometallic Ru catalyst after catalytic experiments applying different amounts of ZnSO_4_, FeSO_4_, and MnSO_4_ as reaction modifiers.

Figure 1 illustrates the XRD patterns of the monometallic Ru catalyst after the catalytic experiments applying different amount of ZnSO_4_, FeSO_4_, and MnSO_4_ as reaction modifiers. As can be observed, characteristic diffractions corresponding to metallic Ru were shown over all measured samples, demonstrating that Ru existed mainly as the metallic state during the hydrogenation reaction. Furthermore, the particle size of the monometallic Ru catalyst before and after catalytic experiments was calculated via the Scherrer equation and is listed in Table 1. In comparison to the fresh sample, no obvious change was observed for Ru particle size after the hydrogenation reaction with different amounts of ZnSO_4_, FeSO_4_, and MnSO_4_ as reaction modifiers. The particle size of all samples ranged from 3.5 nm to 3.7 nm, which was extremely comparable to that of fresh Ru nanoparticle. This suggests that the particle size of Ru was not affected by the utilization of a reaction modifier.

Table 1 gives the composition of the Ru catalyst before and after catalytic experiments as well as the pH value of the slurry. As observed, only Ru was detected over the fresh Ru catalyst and Ru after hydrogenation reaction in pure distilled water. In comparison, Zn, Fe, and Mn were observed when 0.28 mol L^−1^ of ZnSO_4_, FeSO_4_, and MnSO_4_ were applied as reaction modifiers, respectively. Moreover, the molar ratio of added metal to Ru ranged from Fe > Zn > Mn, implying that the ability of adsorption on the Ru surface ranged from FeSO_4_ > ZnSO_4_ > MnSO_4_. Additionally, the pH value of slurry after the catalytic experiments ranged from the same order, demonstrating that MnSO_4_ provided the most acidic condition through its hydrolysis. This suggests that the more easily the salt was hydrolyzed, the more difficult it was to adsorb on the Ru surface. Interestingly, when the concentration of applied reaction modifier was increased to 0.57 mol L^−1^, the amount of adsorbed metal was decreased. This can be rationalized considering that, by enhancing the concentration of the added modifier, the slurry became more acidic due to the hydrolysis of the metal salts, for which the adsorption of the corresponding metal was retarded. This was also confirmed by the pH value of the slurry after the hydrogenation reaction.

**Figure 2:**
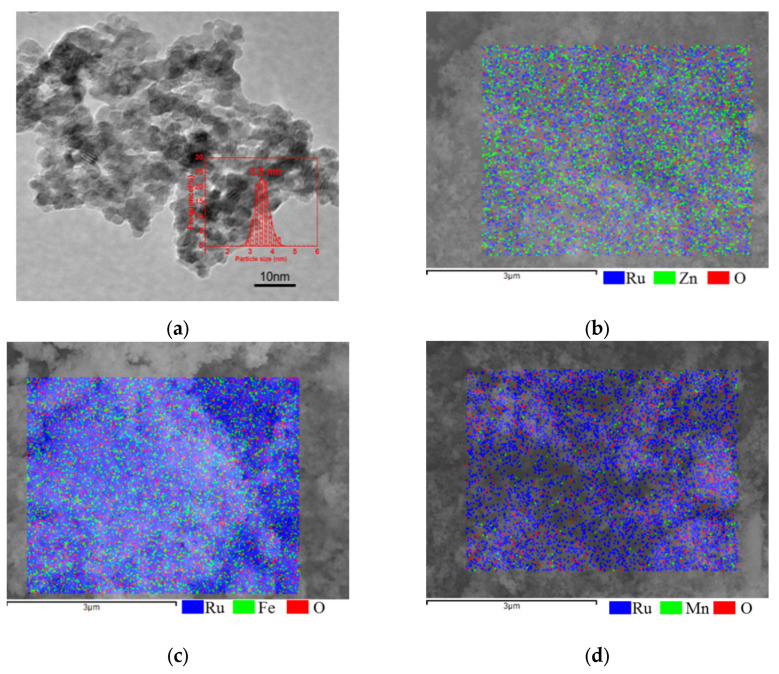
TEM images (**a**) as well as particle size distribution of the fresh Ru nanoparticles (insert image) and the SEM image as well as the element mapping image of Ru catalyst after catalytic experiments adding 0.57 mol L^−1^ of ZnSO_4_ (**b**), FeSO_4_ (**c**), and MnSO_4_ (**d**).

Figure 2 demonstrates the TEM images of the fresh Ru nanoparticles (Figure 2a) and the SEM image as well as the element mapping image of the Ru catalyst after catalytic experiments adding 0.57 mol L^−1^ of ZnSO_4_, FeSO_4_, and MnSO_4_ (Figure 2b–d). As can be seen from Figure 2a, the fresh monometallic Ru catalyst displayed a circular or an elliptical shape. The particle size of Ru was around 3.5 nm, which was in good agreement with XRD results. In addition, it was shown from Figure 2b,c that plenty of Zn and Fe were adsorbed on the Ru surface. On the contrary, only a few Mn were observed from Figure 2c. This was consistent with XRF results (i.e., n(Mn)/n(Ru) was only 0.0023).

**Figure 3:**
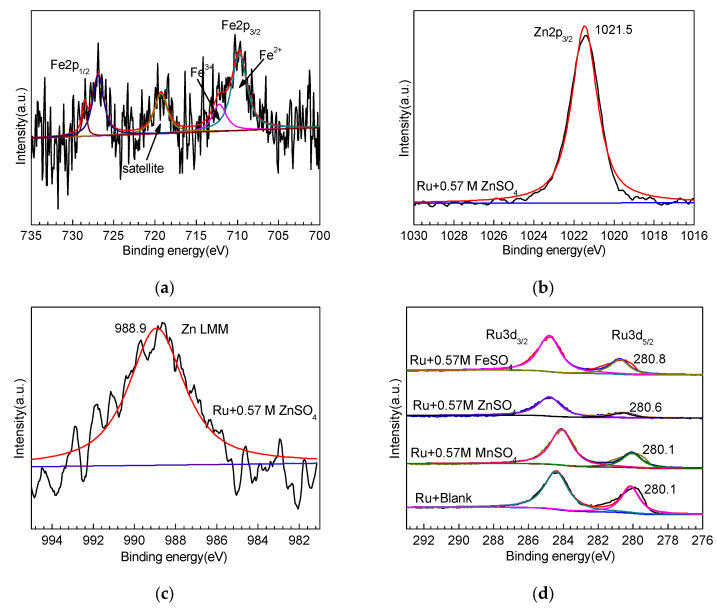
XPS profiles of the monometallic Ru catalyst after hydrogenation reaction applying 0.57 mol L^−1^ of ZnSO_4_, FeSO_4,_ and MnSO_4_ as the reaction modifiers. (**a**) Fe 2p over the monometallic Ru catalyst applying 0.57 mol L^−1^ FeSO_4_; (**b**) Zn 2p_3/2_ over the monometallic Ru catalyst applying 0.57 mol L^−1^ ZnSO_4_; (**c**) Zn LMM over the monometallic Ru catalyst applying 0.57 mol L^−1^ ZnSO_4;_ and (**d**) Ru 3d applying 0.57 mol L^−1^ of ZnSO_4_, FeSO_4_, and MnSO_4_ as the reaction modifiers.

Figure 3 shows the XPS spectra of the monometallic Ru catalyst after hydrogenation reaction applying 0.57 mol L^−1^ of ZnSO_4_, FeSO_4_, and MnSO_4_ as the reaction modifiers, respectively. As can be observed from Figure 3a, two peaks related to binding energies (BE) of 709.8 eV and 712.2 eV were attributed to Fe^2+^ and Fe^3+^, respectively. The peak at 719.3 eV was related to Fe^3+^ [16]. Furthermore, the peak area of Fe^2+^ was significantly larger than that of Fe^3+^, indicating that the adsorbed Fe mainly existed as Fe^2+^. From Figure 3b,c, the BE of Zn 2p_3/2_ and Zn LMM was shown at 1021.5 eV and 988.9 eV, respectively. The BE was the same as Zn^2+^, which was reported by Sun et al. [17,18,19], suggesting that the adsorbed Zn mainly existed as Zn^2+^. Unfortunately, due to the extremely low amount of adsorbed Mn, no reflection attributed to Mn^2+^ was observed. As illustrated in Figure 3d, the BE of Ru 3d_5/2_ was 280.1 eV when no reaction modifier was added, which belonged to metallic Ru. This further proved Ru existed mainly as the metallic state during the hydrogenation reaction. Applying MnSO_4_ as a reaction modifier, the BE of Ru 3d_5/2_ was still observed at 280.1 eV, implying that no electron transfer occurred between Ru and Mn. On the other hand, when ZnSO_4_ and FeSO_4_ were used, the BE of Ru 3d_5/2_ increased from 280.1 eV to 280.6 eV and 280.9 eV, respectively. This can be rationalized by the fact that some electrons were transferred from Ru to Zn and Fe, generating Ru^δ^^+^. In addition, it was deemed that more electrons were transferred from Ru to Fe than from Ru to Zn.

**Figure 4:**
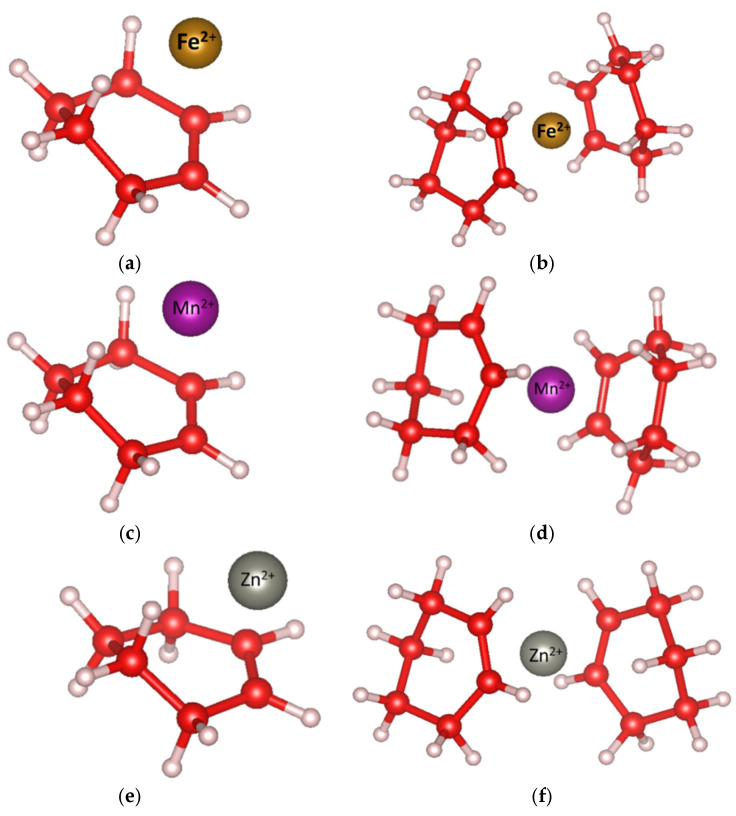
The privileged structure of the formed complex between single/double cyclohexene molecule and Fe^2+^, Mn^2+^, as well as Zn^2+^. (**a**) Fe^2+^ and single cyclohexene molecule; (**b**) Fe^2+^ and double cyclohexene molecules; (**c**) Mn^2+^ and single cyclohexene molecule; (**d**) Mn^2+^ and double cyclohexene molecules; (**e**) Zn^2+^ and single cyclohexene molecule; and (**f**) Zn^2+^ and double cyclohexene molecules.

The privileged structure of the formed complex between single/double cyclohexene molecules and cations is shown in Figure 4, and the corresponding Gibbs free energy (∆_f_*G*) is given in Table 2. As can be seen, the ∆_f_*G* of the formed complex between Mn^2+^ and a single cyclohexene molecule was −1723 kJ mol^−1^, and lower ∆_f_*G* of −1794 kJ mol^−1^ was observed over the complex formed by Fe^2+^ and a single cyclohexene molecule. The most negative ∆_f_*G* of −1866 kJ mol^−1^ was obtained over the complex formed between Zn^2+^ and a single cyclohexene molecule. Furthermore, the same trend of ∆_f_*G* was noticed over the formed complex between two cyclohexene molecules and the cations. This showed that Zn^2+^ was the most suitable cation to stabilize cyclohexene by forming a complex.

**Figure 5:**
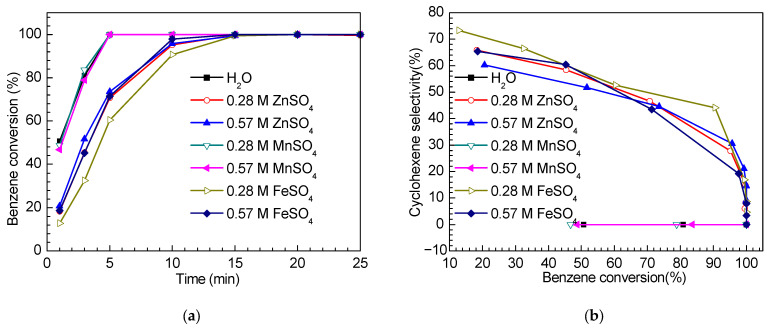
Catalytic activity towards benzene conversion and cyclohexene selectivity over the monometallic Ru catalyst with different reaction modifiers (m_cat_ = 1.8 g, V_H2O_ = 280 mL, V_benzene_ = 140 mL, T = 423 K, p H_2_ = 5.0 MPa) (**a**): benzene conversion; (**b**): cyclohexene selectivity.

Catalytic activity towards benzene conversion and cyclohexene selectivity over monometallic Ru catalyst with different reaction modifiers are illustrated in Figure 5. Notably, complete conversion of benzene was observed within 5 min when MnSO_4_ (both 0.28 and 0.57 mol L^−1^) was applied, and no cyclohexene was detected. Similar results were obtained when no reaction modifier was added, where 100% of benzene was converted to cyclohexane within 5 min of reaction time. In comparison, when ZnSO_4_ or FeSO_4_ were used, catalytic activity towards benzene conversion over monometallic Ru was retarded, while cyclohexene selectivity was increased. With increasing the concentration of used reaction modifier (e.g., ZnSO_4_ or FeSO_4_), catalytic activity towards benzene conversion was enhanced and selectivity to cyclohexene dropped. In addition, higher benzene conversion and lower cyclohexene selectivity was observed with applying ZnSO_4_ than that obtained with utilizing FeSO_4_ as the reaction modifier. Based on the characterization results, it is deemed that the effect of reaction modifier is mainly attributed to the adsorption of cation. When MnSO_4_ is added, Mn^2+^ was barely adsorbed on the Ru surface, thus the catalytic activity and selectivity towards cyclohexene formation was not influenced. Additionally, the amount of adsorbed Zn^2+^ or Fe^2+^ was declined with raising the concentration of applied ZnSO_4_ or FeSO_4_, respectively. This leads to the higher catalytic activity towards benzene conversion and lower selectivity towards cyclohexene generation. How the adsorbed Zn^2+^ and Fe^2+^ affecting the partial hydrogenation of benzene over Ru catalysts can be addressed as following: 1. some electrons could be transferred from Ru to Zn^2+^/Fe^2+^ to generate Ru^δ^^+^, which is less active for activation the π orbital of benzene. Only two of the π orbital of benzene was activated, resulting in the synthesis of cyclohexene via a partial hydrogenation procedure [20]; 2. more stabilized complex could be formed between Zn^2+^/Fe^2+^ and cyclohexene, improving the selectivity towards cyclohexene production [21,22]; 3. the adsorbed Zn^2+^ and Fe^2+^ could cover parts of the active sites of Ru for H_2_ dissociation, hence benefiting the cyclohexene formation [9]. Thus, with increasing the concentration of ZnSO_4_/FeSO_4_, the amount of adsorbed Zn^2+^/Fe^2+^ was declined, hence the selectivity towards cyclohexene was decreased. Furthermore, more Fe^2+^ was adsorbed on the Ru surface than that observed for Zn^2+^, leading to more electron transfer and more active sites coverage for Fe than that for Zn. It is therefore lower catalytic activity towards benzene conversion and higher cyclohexene selectivity was achieved over monometallic Ru catalyst with applying FeSO_4_ than using ZnSO_4_.

### 2.2. Ru-Zn Catalyst

**Figure 6:**
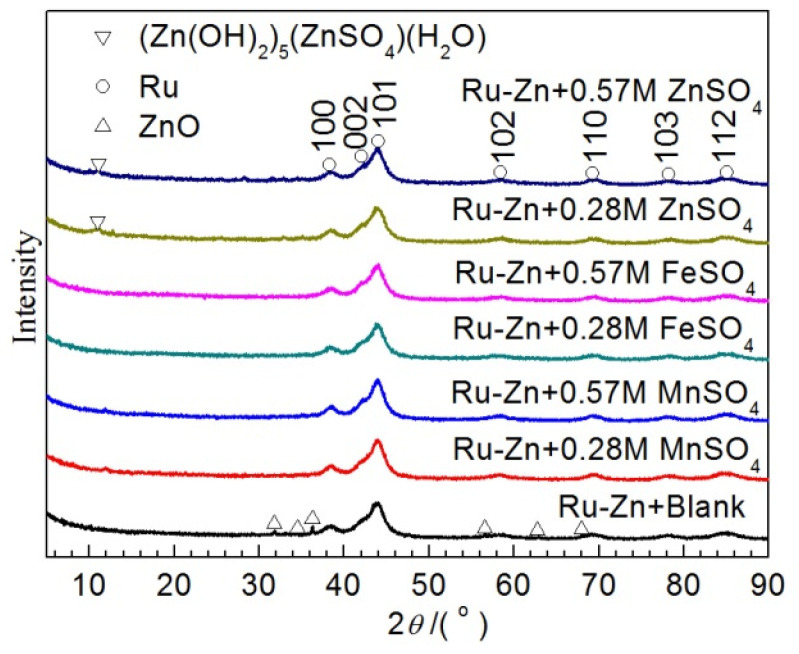
XRD patterns of the Ru-Zn catalyst after catalytic experiments applying different amounts of ZnSO_4_, FeSO_4,_ and MnSO_4_ as reaction modifiers.

Figure 6 exhibits the XRD patterns of the Ru-Zn catalyst after catalytic experiments applying different amounts of ZnSO_4_, FeSO_4_, and MnSO_4_ as reaction modifiers. As can be observed, characteristic diffractions corresponding to metallic Ru with the hexagonal phase and ZnO with the hexagonal phase were observed over Ru-Zn after hydrogenation without adding any reaction modifier, demonstrating that Ru and Zn existed mainly as metallic Ru and ZnO, respectively. Applying MnSO_4_ or FeSO_4_ (both 0.28 and 0.57 mol L^−1^), the reflection to ZnO could no longer be observed. This can be attributed to the fact that MnSO_4_ or FeSO_4_ could react with ZnO to form new compounds, which were dissolvable under the reaction conditions. When ZnSO_4_ was added as a reaction modifier, a new diffraction related to (Zn(OH)_2_)_5_(ZnSO_4_)(H_2_O) was observed, indicating that ZnO could react with ZnSO_4_ to generate (Zn(OH)_2_)_5_(ZnSO_4_)(H_2_O). The same results were reported by Sun et al. [23] and Yan et al. [24].

Table 3 lists the composition and the particle size of the Ru-Zn catalyst after catalytic experiments as well as the pH value of the slurry. Without using any reaction modifier, the molar ratio of Zn to Ru was close to that of the fresh Ru-Zn catalyst (e.g., n(Zn)/n(Ru) = 0.2645), suggesting that Zn was barely lost during the catalytic experiment. The slurry was close to neutral (pH = 6.95). When 0.28 mol L^−1^ of MnSO_4_ was used, n(Zn)/n(Ru) was decreased from 0.2645 to 0.2228, and n(Mn)/n(Ru) was also raised in comparison to that observed over the monometallic Ru catalyst (e.g., 0.0247 vs. 0.0019). This was mainly because the acidic condition was neutralized by ZnO. Hence, the pH value of the slurry increased, resulting in more Mn^2+^ and SO_4_^2−^ being adsorbed on the Ru surface. While the concentration of used MnSO_4_ was increased to 0.57 mol L^−1^, the slurry became more acidic, leading to less adsorption of Zn^2+^, Mn^2+^, and SO_4_^2−^ than that obtained with 0.28 mol L^−1^ of MnSO_4_. Furthermore, with addition of 0.28 mol L^−1^ of ZnSO_4_, the n(Zn)/n(Ru) slightly dropped in comparison to that detected without any reaction modifier (e.g., 0.2360 vs. 0.2645). This can be rationalized considering that part of ZnO was dissolved under such acidic conditions. However, a significant increase of S was observed, i.e., n(S)/n(Ru) = 0.0216, and the molar ratio of Zn to Ru was obviously higher than that observed over the monometallic Ru catalyst under the same conditions (e.g., 0.2360 vs. 0.0307). This was mainly due to the formation of (Zn(OH)_2_)_5_(ZnSO_4_)(H_2_O) salt from the reaction between ZnO and ZnSO_4_, which benefited the adsorption of Zn^2+^. By enhancing the concentration of ZnSO_4_ from 0.28 mol L^−1^ to 0.57 mol L^−1^, the pH value decreased from 6.08 to 5.61. This resulted in the lower content of formed (Zn(OH)_2_)_5_(ZnSO_4_)(H_2_O), hence the adsorbed Zn^2+^. On the other hand, when FeSO_4_ was applied, a severe decline of adsorbed Zn was noticed. It was deemed that FeSO_4_ could react with ZnO to form salt-like basic Fe sulfate salt, which depressed the adsorption of Zn. Moreover, the particle size of all Ru-Zn catalysts ranged from 3.7 nm to 3.9 nm after the experiments, suggesting that particle size of Ru-Zn was not affected by the utilization of reaction modifiers.

**Figure 7:**
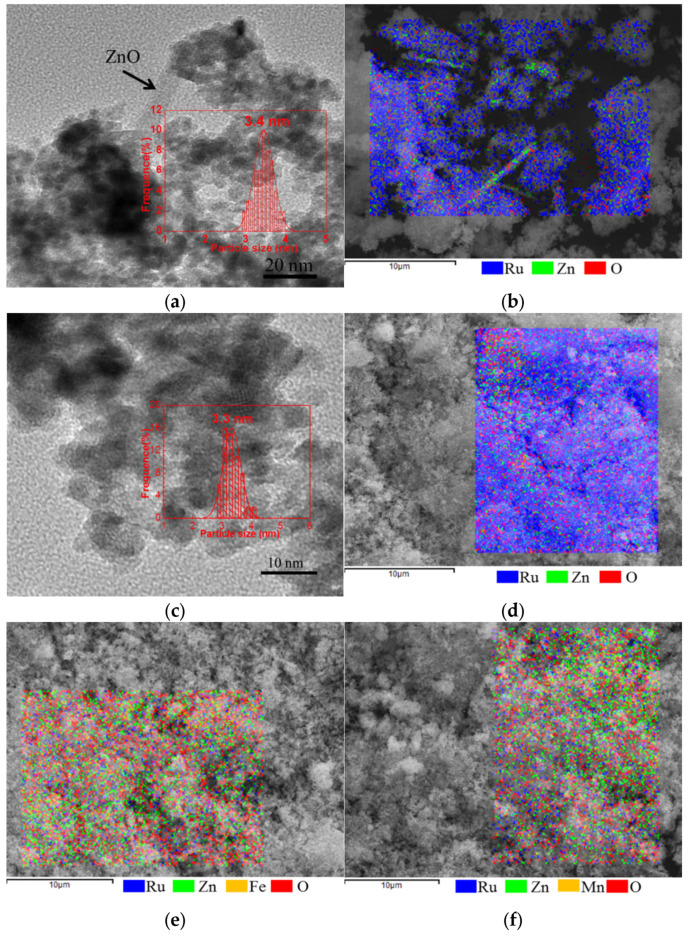
TEM images, SEM images, particle size distribution, and element mapping image of the spent Ru-Zn catalyst with different reaction modifiers added. (**a**) TEM image and particle size distribution (insert image) of the spent Ru-Zn catalyst without using any reaction modifier; (**b**) SEM image as well as element mapping image of the spent Ru-Zn catalyst without using any reaction modifier; (**c**) TEM image of the spent Ru-Zn catalyst applying ZnSO_4_ (0.57 mol L^−1^) as the reaction modifier; (**d**) SEM image as well as the element mapping image of the spent Ru-Zn catalyst using ZnSO_4_ as a reaction modifier; (**e**) SEM image as well as the element mapping image of the spent Ru-Zn catalyst using FeSO_4_ as a reaction modifier; (**f**) SEM image as well as the element mapping image of the spent Ru-Zn catalyst using MnSO_4_ as a reaction modifier.

The TEM and the SEM images as well as the element mapping image of the spent Ru-Zn catalyst without using any reaction modifier are illustrated in Figure 7a,b, respectively. It can be seen from Figure 7a that the spent Ru-Zn catalyst displayed a circular or an elliptical shape, of which particle size was around 3.4 nm. Moreover, rodlike ZnO was observed, indicating that ZnO could not be uniformly dispersed on the surface of the catalyst. As observed from Figure 7b, the rodlike shape of the Zn element was also observed, which was consistent with the TEM results. Figure 7c displays the TEM image of the spent Ru-Zn catalyst applying ZnSO_4_ (0.57 mol L^−1^) as the reaction modifier. No rodlike ZnO was detected, suggesting that ZnO was consumed through the reaction with ZnSO_4_. Furthermore, the particle size of Ru-Zn was mainly distributed at 3.3 nm, which was comparable to that obtained over spent Ru-Zn without using any reaction modifier. This implied that the particle size of Ru-Zn could not be affected by utilization of the reaction modifier. Figure 7d demonstrates the SEM image as well as the element mapping image of the spent Ru-Zn catalyst using ZnSO_4_ as a reaction modifier. No rodlike ZnO could be observed, and Zn species was uniformly dispersed on the surface of the Ru-Zn catalyst. This can be rationalized considering that (Zn(OH)_2_)_5_(ZnSO_4_)(H_2_O) could be generated and uniformly dispersed on the surface of the Ru-Zn catalyst. When FeSO_4_ was added (0.57 mol L^−1^), as shown in Figure 7e, both Zn and Fe species were uniformly dispersed on the surface of the Ru-Zn catalyst. Besides, rodlike ZnO disappeared. A similar phenomenon was observed when MnSO_4_ (0.57 mol L^−1^) was applied, where Zn and Mn species were also uniformly dispersed on the surface of the Ru-Zn catalyst. Interestingly, more Mn was observed in comparison to that detected over the monometallic Ru catalyst, indicating an enhancement of adsorbed Mn on the Ru-Zn surface.

**Figure 8:**
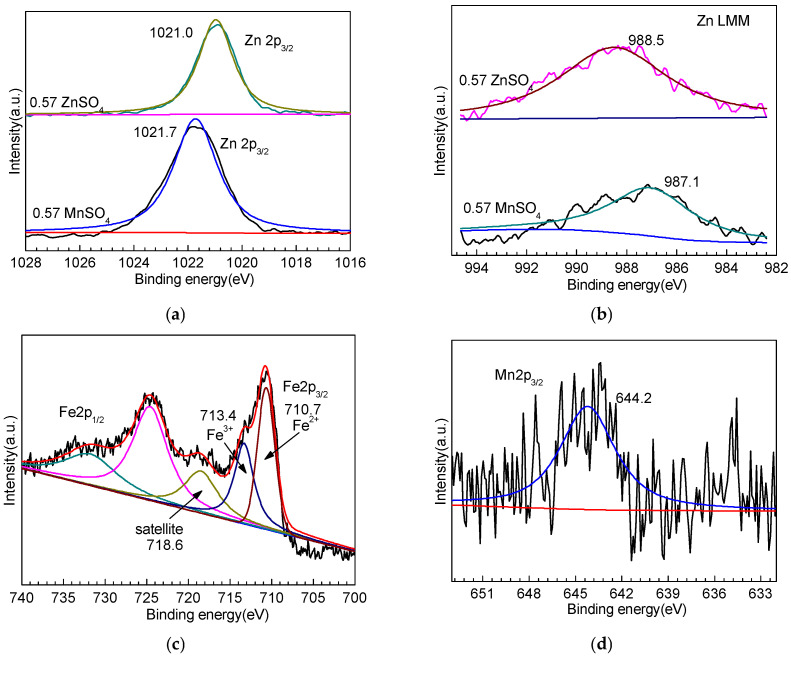

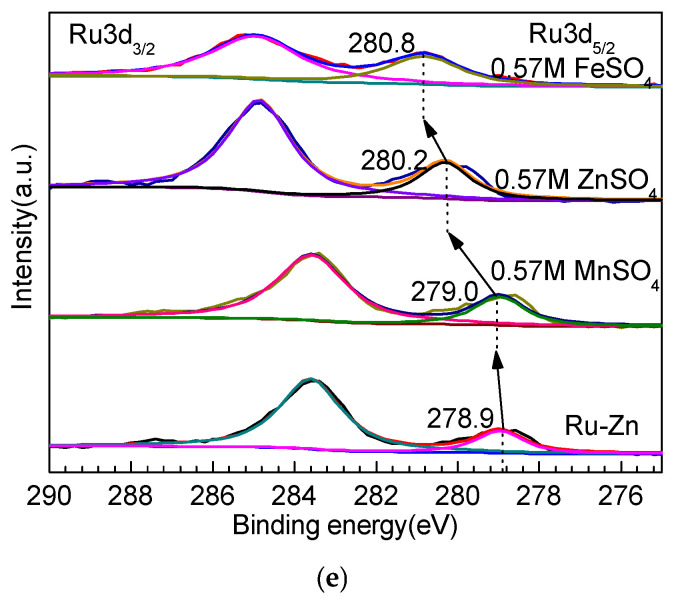
XPS profiles of the Ru-Zn catalyst after hydrogenation reaction applying 0.57 mol L^−1^ of ZnSO_4_, FeSO_4,_ and MnSO_4_ as the reaction modifiers. (**a**) Zn 2p_3/2_ over the Ru-Zn catalyst applying 0.57 mol L^−1^ of ZnSO_4_ and MnSO_4_, respectively; (**b**) Zn LMM over the Ru-Zn catalyst applying 0.57 mol L^−1^ ZnSO_4_ and MnSO_4_, respectively; (**c**) Fe 2p over the Ru-Zn catalyst applying 0.57 mol L^−1^ FeSO_4_; (**d**) Mn 2p_3/2_ over the Ru-Zn catalyst applying 0.57 mol L^−1^ MnSO_4;_ and (**e**) Ru 3d_5/2_ with and without reaction modifiers.

Figure 8 shows the XPS profiles of the Ru-Zn catalyst after catalytic experiments applying ZnSO_4_, FeSO_4_, and MnSO_4_ (all 0.57 mol L^−1^) as the reaction modifier, respectively. The BE of Zn 2p_3/2_ and Zn LMM over Ru-Zn catalyst using ZnSO_4_ or MnSO_4_ as reaction modifier was demonstrated in Figure 8a,b. With addition of ZnSO_4_, the BEs of Zn 2p_3/2_ and Zn LMM over Ru-Zn catalyst were observed at 1021.0 eV and 988.5 eV, respectively. In comparison, when MnSO_4_ was applied, the BEs of Zn 2p_3/2_ and Zn LMM over the Ru-Zn catalyst were observed at 1021.7 eV and 987.1 eV, respectively. It was deemed that Zn mainly existed as Zn^2+^, as the BE of Zn LMM for metallic Zn was 990.1 eV [25]. More importantly, more electrons were transferred from Zn to Ru using ZnSO_4_ than those obtained using MnSO_4_ as the reaction modifier. As can be observed from Figure 8c, two peaks related to binding energies of 710.7 eV and 713.4 eV were attributed to Fe^2+^ and Fe^3+^, respectively. Besides, the satellite diffraction at 718.6 eV belonged to Fe^2+^ [26]. Furthermore, the peak area of Fe^2+^ was obviously larger than that of Fe^3+^, suggesting that the adsorbed Fe mainly existed as Fe^2+^. As observed in Figure 8d, the BE of Mn 2p_3/2_ was shown at 644.2 eV, which was attributed to Mn^2+^ [27]. This implied that the adsorbed Mn was not reduced. Furthermore, as illustrated in Figure 8e, the BE of Ru 3d_5/2_ was 278.9 eV when no reaction modifier was added, which belonged to metallic Ru. This revealed that undispersed ZnO of Ru-Zn could not affect the electronic structure of Ru. Applying MnSO_4_ as a reaction modifier, the BE of Ru 3d_5/2_ was slightly increased from 278.9 eV to 279.0 eV. Based on the aforementioned discussion, it was already concluded that adsorbed Mn could not affect the electronic structure of Ru. Therefore, combined with the shift for BE of Zn 2p_3/2_ and Zn LMM, it was deemed that the slight raise for the BE of Ru 3d_5/2_ was attributed to the adsorbed Zn. In addition, an obvious increase of the BE of Ru 3d_5/2_ was noticed when ZnSO_4_ was applied, i.e., from 278.9 eV to 280.2 eV. This can be rationalized in terms of the formation of (Zn(OH)_2_)_5_(ZnSO_4_)(H_2_O), which contained more Zn species. Higher amounts of adsorbed Zn resulted in the higher BE of Ru 3d_5/2_, implying that more Ru^δ+^ was generated. Moreover, the highest BE of Ru 3d_5/2_ was observed with addition of FeSO_4_ (e.g., 280.8 eV), demonstrating that the most electrons were transferred from Ru to Fe.

**Figure 9:**
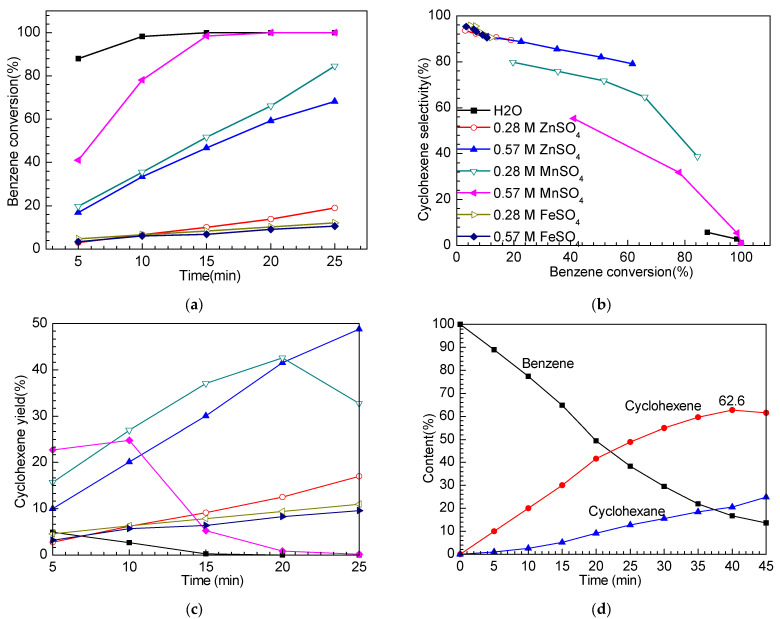
Catalytic activity towards benzene conversion and cyclohexene selectivity over the Ru-Zn catalyst with different reaction modifiers (m_cat_ = 1.8 g, V_H2O_ = 280 mL, V_benzene_ = 140 mL, T = 423 K, p H_2_ = 5.0 MPa) (**a**): benzene conversion; (**b**): cyclohexene selectivity; (**c**) cyclohexene yield; and (**d**) kinetic curve over Ru-Zn with addition of 0.57 mol L^−1^ of ZnSO_4_.

Catalytic activity towards benzene conversion and cyclohexene selectivity over the Ru-Zn catalyst with different reaction modifiers are illustrated in Figure 9. As can be observed from Figure 9a,b, in comparison to that achieved with no reaction modifier, a significant decrease of catalytic activity towards benzene conversion as well as an increase towards cyclohexene formation were obtained by using reaction modifiers. Among the used reaction modifiers, the lowest benzene conversion and the highest cyclohexene selectivity were achieved over the Ru-Zn catalyst using 0.57 mol L^−^^1^ of FeSO_4_, and 0.28 mol L^−^^1^ of MnSO_4_ gave the highest benzene conversion and the lowest cyclohexene selectivity within 25 min of reaction time. Notably, a lower concentration of ZnSO_4_/MnSO_4_ and a higher concentration of FeSO_4_ were beneficial for hindering the catalytic activity towards benzene conversion and improving the selectivity towards cyclohexene generation over the Ru-Zn catalyst. Based on the characterization results and discussions, it was deemed that the adsorbed Zn^2+^/Fe^2+^ played the essential role in the synthesis of cyclohexene from partial hydrogenation of benzene over the Ru-Zn catalyst. This can be rationalized considering that some electrons could be transferred from Ru to Zn^2+^/Fe^2+^ to generate Ru^δ^^+^, which could only activate two of the π orbital of benzene, resulting in benefits for synthesis of cyclohexene via a partial hydrogenation procedure. Furthermore, Zn^2+^/Fe^2+^ was of great capability to stabilize cyclohexene, for which the further hydrogenation of cyclohexene to cyclohexane was retarded. Additionally, the adsorbed Zn^2+^ and Fe^2+^ could cover parts of the most active sites of Ru for direct hydrogenation of benzene to cyclohexane, thus benefiting the formation of cyclohexene. Considering both catalytic activity towards benzene conversion and selectivity to cyclohexene, ZnSO_4_ with a concentration of 0.57 mol L^−^^1^, among all investigated reaction modifiers, was the most suitable for partial hydrogenation of benzene for cyclohexene production over Ru-Zn catalysts. As observed from Figure 9c,d, 48.1% of cyclohexene yield was achieved over the Ru-Zn catalyst applying 0.57 mol L^−^^1^ of ZnSO_4_ as a reaction modifier within 25 min of the catalytic experiment, and the highest cyclohexene yield of 62.6% was obtained when the reaction time reached 40 min, which was one of the highest yields of cyclohexene to ever be reported [4,28].

## 3. Conclusions

Nano Ru-based catalysts, including monometallic Ru and Ru-Zn nanoparticles, were synthesized and evaluated on partial hydrogenation of benzene towards cyclohexene generation, during which the effect of the reaction modifiers, i.e., ZnSO_4_, MnSO_4_, and FeSO_4_, was investigated. It was found that Zn^2+^ or Fe^2+^ could be adsorbed on the surface of the monometallic Ru catalyst, which could stabilize the formed cyclohexene. This led to an enhancement of catalytic selectivity towards cyclohexene. Furthermore, electron transfer was observed from Zn^2+^ or Fe^2+^ to Ru, hindering the catalytic activity towards benzene conversion. In comparison, very few Mn^2+^ cations were adsorbed on the Ru surface, for which no cyclohexene could be detected. On the other hand, for the Ru-Zn catalyst, Zn existed as rodlike ZnO. The added ZnSO_4_ and FeSO_4_ could react with ZnO to generate (Zn(OH)_2_)_5_(ZnSO_4_)(H_2_O) and Fe(OH)SO_4_, respectively. This further benefited the adsorption of Zn^2+^ or Fe^2+^, leading to the decrease of catalytic activity towards benzene conversion and the increase of selectivity towards cyclohexene synthesis. When 0.57 mol L^−^^1^ of ZnSO_4_ was applied, the highest cyclohexene yield of 62.6% was achieved. When MnSO_4_ was used as a reaction modifier, H_2_SO_4_ could be generated in the slurry via its hydrolysis, which reacted with ZnO to form ZnSO_4_. The selectivity towards cyclohexene formation was then improved by the adsorbed Zn^2+^.

## 4. Materials and Methods

### 4.1. Chemicals

All regents were analytical grade. RuCl_3_·3H_2_O was received from Sino-Platinum Co. Ltd. (Kunming, China). NaOH, FeSO_4_·7H_2_O, ZnSO_4_·7H_2_O, MnSO_4_·H_2_O, and benzene were purchased from Kemiou Chemical Reagent Co. Ltd. (Tianjin, China). All chemicals were directly used without any further purification, and deionized water was utilized in all experiments.

### 4.2. Preparation of Catalysts

Ru-Zn catalysts were synthesized via the following procedure: 100 mL of NaOH (0.75 mol·L^−1^) aqueous solution was added into 100 mL aqueous solution of RuCl_3_ (0.18 mol·L^−1^) and ZnSO_4_ (0.06 mol L^−1^) with constant stirring at 353 K for 30 min. Then, the solid part was washed until reaching pH = 7. After that, the solid was dispersed in 200 mL of deionized water, followed by a reducing procedure under 5.0 MPa of H_2_ in a 1000 mL Hastelloy autoclave with a stirring speed of 800 rpm at 423 K. After 3 h of reduction and cooling to 298 K, the fresh catalysts were gained by washing to neutral and were vacuum-dried. The obtained Ru-Zn catalysts were denoted as Ru-Zn. Similarly, monometallic Ru catalysts were synthesized from the same procedure without introducing ZnSO_4_·7H_2_O.

### 4.3. Catalytic Experimental Procedure

All catalytic experiments took place in a 1000 mL GS-1 type Hastelloy autoclave. In a typical reaction, a Ru-Zn catalyst with 0.57 mol·L^−1^ ZnSO_4_ as an instance as well as a 1.8 g Ru-Zn catalyst and a 280 mL aqueous solution of 0.57 mol·L^−1^ ZnSO_4_ were poured into the autoclave. Then, the reactor was purified with N_2_ by increasing the pressure to 5 MPa and depressurizing to 0.2 MPa 4 times. This was followed by purification using H_2_ another 4 times with the same procedure. Then, the autoclave was heated under 5.0 MPa of H_2_ with a stirring speed of 800 rpm. When the temperature reached 423 K, 140 mL of benzene was added into the reactor, and the stirring speed was adjusted to be 1400 rpm to eradicate the effect of mass transfer limitation. Subsequently, the liquid samples were taken from the autoclave every 5 min. All withdrawn samples were analyzed by GC-FID. The benzene conversion and the selectivity towards cyclohexene were calculated with the calibration area normalization method. After each reaction, the catalyst sample was washed until neutral and dried in an Ar atmosphere at 373 K, then stored in ethanol for further characterization. Monometallic Ru and Ru-Zn catalysts with different reaction modifiers were evaluated with the same procedure. In addition, it is important to mention that the mass balance of the reaction was 100% closed.

### 4.4. Catalysts Characterization

X-ray diffraction (XRD) patterns were obtained at the range of 2θ from 5° to 90°, with a step size of 0.03° using diffracted intensity of Cu-Kα radiation (λ = 0.15418 nm) via an X’Pert Pro instrument (PAN Nallytical, Almelo, The Netherlands). Scherrer’s equation was applied for the calculation of the particle size of tested samples. In addition, elemental analysis was conducted via X-ray fluorescence (XRF) (S4 Pioneer instrument from Bruker AXS, Karlsruhe, Germany). Furthermore, the morphology of the catalyst surface as well as the element scanning was tested by transmission electron microscope (TEM), a JEOL JEM 2100 from Akishima, Japan. Surface composition of the selected samples was identified by energy dispersive spectrometer (EDS). Moreover, the valence states of Ru, Zn, Fe, and Mn as well as the kinetic energy of Zn LMM electrons on the catalyst surface were analyzed by X-ray photoelectron spectroscopy (XPS) using a PHI Quantera SXM instrument from ULVAC-PHI, Japan. Al Kα (Eb = 1486.6 eV) was chosen as the radiation source, and the vacuum degree was adjusted to be 6.7 × 10^‒8^ Pa. The C1s (Eb = 284.8 eV) line as the binding energy reference was used for calibrating and correcting the energy scale.

### 4.5. Theoretical Calculation

The B3LYP density functional method in conjunction with the 6-31G(d,p) basis set for cyclohexene and Lanl2DZ for the metal ions was used to optimize the complexes formed between cyclohexene and the metal ions of Fe^2+^, Mn^2+^, and Zn^2+^. The calculations were all performed with the Gaussian 09 software package. Vibrational frequency analyses were performed for the optimized geometries.

## Figures and Tables

**Table 1:** Composition and particle size of the monometallic Ru before and after catalytic experiments with different amounts of reaction modifiers as well as the pH value of slurry.

Conditions	*n*(Zn)/*n*(Ru) ^1^ (mol/mol)	*n*(Mn)/*n*(Ru) ^1^ (mol/mol)	*n*(Fe)/*n*(Ru) ^1^ (mol/mol)	Particle Size ^2^ (nm)	pH Value ^3^
Ru catalyst	-	-	-	3.6	-
Ru + Blank	-	-	-	3.6	6.95
Ru + 0.28 M MnSO_4_	-	0.0019	-	3.7	3.23
Ru + 0.57 M MnSO_4_	-	0.0023	-	3.6	3.11
Ru + 0.28 M FeSO_4_	-	-	0.0358	3.5	4.66
Ru + 0.57 M FeSO_4_	-	-	0.0295	3.7	3.83
Ru + 0.28 M ZnSO_4_	0.0307	-	-	3.6	4.35
Ru + 0.57 M ZnSO_4_	0.0258	-	-	3.7	3.53

^1^ Determined by XRF instrument; ^2^ determined by XRD instrument; ^3^ pH value of the slurry after catalytic experiments at room temperature.

**Table 2:** The Gibbs free energy of the formed complex between single/double cyclohexene molecules and cations.

Complex	∆_f_*G*/(kJ∙mol^−1^)
Cyclohexene Mn(Ⅱ)	−1723
(Cyclohexene)_2_Mn(Ⅱ)	−1598
Cyclohexene Fe(Ⅱ)	−1794
(Cyclohexene)_2_Fe(Ⅱ)	−1661
Cyclohexene Zn(Ⅱ)	−1866
(Cyclohexene)_2_Zn(Ⅱ)	−1815

**Table 3:** Composition and particle size of monometallic Ru before and after catalytic experiments with different amounts of reaction modifiers as well as the pH value of slurry.

Conditions	n(Zn)/n(Ru) ^1^ (mol/mol)	n(Mn)/n(Ru) ^1^ (mol/mol)	n(Fe)/n(Ru) ^1^ (mol/mol)	n(S)/n(Ru) (mol/mol)	Particle ^2^ Size(nm)	pH ^3^ Value
Ru-Zn(0.27) + Blank	0.2645	-	-	-	3.7	6.95
Ru-Zn(0.27) + 0.28 M MnSO_4_	0.1528	0.0247	-	0.0078	3.8	6.98
Ru-Zn(0.27) + 0.57 M MnSO_4_	0.1413	0.0225	-	0.0053	3.9	6.85
Ru-Zn(0.27) + 0.28 M FeSO_4_	0.0624	-	0.2422	0.0032	3.7	6.92
Ru-Zn(0.27) + 0.57 M FeSO_4_	0.0542	-	0.2782	0.0048	3.8	6.51
Ru-Zn(0.27) + 0.28 M ZnSO_4_	0.2360	-	-	0.0216	3.8	6.08
Ru-Zn(0.27) + 0.57 M ZnSO_4_	0.2135	-	-	0.0183	3.9	5.61

^1^ Determined by XRF instrument; ^2^ determined by XRD instrument; ^3^ pH value of the slurry after catalytic experiments at room temperature.

## Data Availability

The data presented in this study are available in this work.
